# Conditionally-live attenuated SIV upregulates global T effector memory cell frequency under replication permissive conditions

**DOI:** 10.1186/1742-4690-10-59

**Published:** 2013-06-05

**Authors:** Maria S Manoussaka, Neil Berry, Deborah Ferguson, Richard Stebbings, Mark Robinson, Claire Ham, Mark Page, Bo Li, Atze T Das, Ben Berkhout, Neil Almond, Martin P Cranage

**Affiliations:** 1 Centre for Infection & Immunity, Division of Clinical Sciences, St George’s, University of London, Cranmer Terrace, London, SW17 0RE, United Kingdom; 2 Divisions of Virology & Biotherapeutics, National Institute for Biological Standards and Control, Potters Bar, UK; 3 Laboratory of Experimental Virology, Academic Medical left of the University of Amsterdam, Amsterdam, The Netherlands

**Keywords:** Live attenuated SIV, T effector memory, Vaccine

## Abstract

**Background:**

Live attenuated SIV induces potent protection against superinfection with virulent virus; however the mechanism of this vaccine effect is poorly understood. Such knowledge is important for the development of clinically acceptable vaccine modalities against HIV.

**Results:**

Using a novel, doxycycline dependent, replication-competent live-attenuated SIVmac239Δ*nef* (SIV-rtTAΔ*nef*), we show that under replication-permissive conditions SIV-rtTAΔ*nef* is fully viable. Twelve rhesus macaques were infected with a peak plasma vRNA on average two log_10_ lower than in 6 macaques infected with unconditionally replication-competent SIVΔ*nef*. Consistent with the attenuated phenotype of the viruses the majority of animals displayed low or undetectable levels of viraemia by 42-84 days after infection. Next, comparison of circulating T cells before and after chronic infection with parental SIVΔ*nef* revealed a profound global polarisation toward CD28^-^CCR7^-^ T-effector memory 2 (T_EM2_) cells within CD95^+^CD4^+^ and CD95^+^CD8^+^ populations. Critically, a similar effect was seen in the CD95^+^ CD4^+^ population and to somewhat lesser extent in the CD95^+^ CD8^+^ population of SIV-rtTAΔ*nef* chronically infected macaques that were maintained on doxycycline, but was not seen in animals from which doxycycline had been withdrawn. The proportions of gut-homing T-central memory (T_CM_) and T_EM_ defined by the expression of α4β7 and CD95 and differential expression of CD28 were increased in CD4 and CD8 cells under replication competent conditions and gut-homing CD4 T_CM_ were also significantly increased under non-permissive conditions. T_EM2_ polarisation was seen in the small intestines of animals under replication permissive conditions but the effect was less pronounced than in the circulation. Intracellular cytokine staining of circulating SIV-specific T cells for IL-2, IFN-γ, TNF-α and IL-17 showed that the extent of polyfunctionality in CD4 and CD8 T cells was associated with replication permissivity; however, signature patterns of cytokine combinations were not distinguishable between groups of macaques.

**Conclusion:**

Taken together our results show that the global T memory cell compartment is profoundly skewed towards a mature effector phenotype by attenuated SIV. Results with the replication-conditional mutant suggest that maintenance of this effect, that may be important in vaccine design, might require persistence of replicating virus.

## Background

Because of the predilection of human and simian immunodeficiency viruses to replicate in cells of the immune system and particularly in CD4 T cells it is an extremely complex task to unravel the exact relationships between viral dynamics, influenced by virus and host phenotype and the cognate adaptive and innate immune responses. These responses are themselves influenced directly by virus replication and bystander effects driven by inappropriate immune activation and immunosuppression. It is however clear that T cell immune responses have a profound influence on on-going virus replication as evidenced by (1) the temporal relationship between their appearance and the down-turn in concentrations of plasma vRNA [1,2], (2) the strong selective pressure exerted on T cell epitope recognition [3,4] and (3) experiments in SIV-infected macaques where selective depletion of CD8 T cells correlated with reduced virus control [5,6]. Conversely, the generation of T cell responses is driven by virus replication and antigen expression which themselves are modulated by the effects of the virus on the integrity of the immune system including continuous immune activation characterised by increased T cell turnover, the production of pro-inflammatory cytokines and distinct B cell dysfunction [7-9]. Moreover, it is now realised that infection has a profound and early impact on the gut epithelial barrier associated with local inflammation and loss of integrity of the mucosal immune system. Since the gut accommodates a rich environment of microbes and microbial products, its damage may cause increased permeability and translocation of microbial products into the periphery fuelling continuous systemic immune activation, a hallmark of HIV/SIV pathogenesis [10,11].

The use of SIV mutants in the macaque model allows the dissection of the complex interplay between the host and virally-encoded pathogenesis factors and has been critical in revealing the ability of attenuated virus to induce a state of superinfection resistance *in vivo*. It is well established that removal or interruption of the viral accessory gene *nef* attenuates HIV and SIV *in vivo* resulting in the early acute phase viraemia progressing to a very low set-point where virus rarely if ever is detected in the peripheral circulation either by virus isolation from peripheral blood mononuclear cells (PBMC) or by RT-PCR amplification of vRNA [12,13]. This attenuated peripheral phenotype of viral replication is associated with the generation of CD8 and CD4 T cell responses which are widely disseminated and detectable at mucosal sites regardless of the virus portal of entry. Moreover, macaques infected with attenuated SIV display potent resistance to subsequent superinfection challenge with cell-free homologous and heterologous viruses including chimeric SIV expressing HIV envelope [14-16] and virus infected cells [17]. Furthermore, protection extends to mucosal challenge [16,18,19]. Even when superinfection does occur, disease progression appears to be ameliorated by the effects of the pre-existing attenuated virus [12]. Although, taken together, these findings suggest that live attenuated vaccination would be an approach to vaccination against HIV safety issues including reversion to virulence by mutation [20] and differential pathogenicity dependent upon host factors [21] have precluded direct development of this strategy. Nonetheless, mechanistic insight into this powerful effect will inform rational design of clinically acceptable vaccines.

To more fully understand the live attenuated vaccine effect it is imperative to define the parameters required for protection. As for other attenuated virus vaccines it is known that protection is influenced by the degree of attenuation, as reflected in the acute peak of plasma viraemia [22,23]. However, less is known about events following clearance of attenuated virus from the peripheral circulation. In this study we were interested to determine the attenuated vaccine-driven T cell environment and cognate T cell responses under conditions where on-going replication in tissues (occult replication) was permitted compared to non-replication permissive conditions. To address this issue we have used a novel conditionally replication competent variant of SIVmac239Δ*nef* (SIVΔ*nef*): a prototypic attenuated SIV that confers potent protection against superinfection [12]. The conditional mutant, designated SIVmac239rtTAΔ*nef* (SIV-rtTAΔ*nef*), replicates exclusively when doxycycline (dox) is administered *in vitro*[24,25].

We show here in rhesus macaques that SIV-rtTAΔ*nef* replicated *in vivo* in the presence of orally administered dox and drives polarisation of the global circulating T cell memory compartment toward a T_EM_ phenotype, most notably in the fully differentiated T_EM2_ population (CD95^+^CD28^-^CCR7^-^). A similar effect was seen in SIVΔ*nef* -infected macaques. Critically this phenotype was not seen following withdrawal of dox in SIV-rtTAΔ*nef* -infected macaques *i.e.* under replication non-permissive conditions. Maintenance of gut-homing α4^+^β7^+^ T_EM_ (CD95^+^CD28^-^) also was dependent on replication permissivity whereas increased proportions of CD4 and CD8 α4^+^β7^+^ T_CM_ (CD95^+^CD28^+^) were observed in SIV-rtTAΔ*nef* -infected macaques in both the continued presence of dox and following withdrawal of dox. Analysis of small intestine tissues demonstrated marked differences in vRNA and Env antigen staining intensity and distribution between macaques infected with SIV-rtTAΔ*nef* under replication permissive and non-permissive conditions and animals infected with SIVΔ*nef*; however, polarisation of global T cell memory phenotypes was less pronounced in cells extracted from small intestine. Analysis of SIV-antigen driven T cell polyfunctionality revealed no clearly defined IL-2, IFN-γ, TNF-α and IL-17 signature with respect to virus replication dynamics at the level of individual cytokine combinations in either CD4 or CD8 circulating or small intestinal T cell populations; however, the degree of polyfunctionality was related to replicative capacity and replication permissivity.

## Results

### SIV-rtTAΔ*nef* had an attenuated phenotype *in vivo* with reduced acute phase replication compared to SIVΔ*nef*

**Figure 1:**
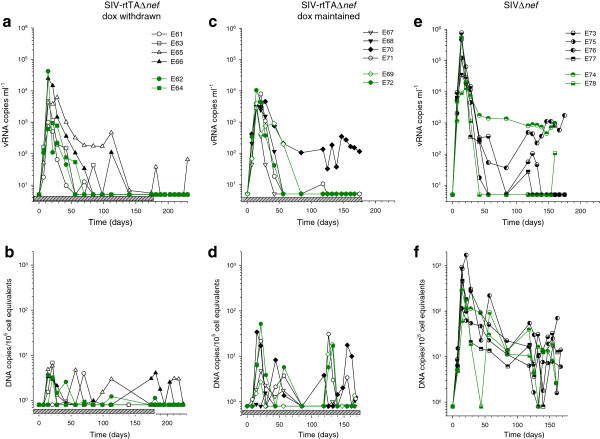
**Comparative viral replication dynamics.** Plasma viraemia and proviral DNA loads in PBMC were measured by quantative real-time RT-PCR and quantative PCR respectively over time in rhesus macaques infected with either SIV-rtTAΔ*nef* where dox was withdrawn after 175 days (Group A) (**a** &**b**), or where dox was maintained for the duration of the analysis (Group B) (**c** &**d**) or infected with SIVΔ*nef* (Group C) (**e** &**f**). The grey stippled bars indicate the period of dox administration. Green lines and symbols show animals that were also analysed for virological and immunological parameters in tissues.

A total of 12 macaques, pre-dosed with dox for 14 days were inoculated with SIV-rtTAΔ*nef* and maintained on dox for a further 175 days (Groups A & B). Six animals were followed for a further 56 days following withdrawal of dox (Group A) and a further 6 animals were inoculated with SIVΔ*nef* in the absence of dox (Group C). All animals were successfully infected resulting in median peak plasma vRNA loads of 4.34 × 10^3^ (range 9.44 × 10^2^ – 2.49 × 10^4^) vRNA copies/ml for SIV-rtTAΔ*nef* and 2.95 × 10^5^ (range 1.32 × 10^4^ – 7.73 × 10^5^) vRNA copies/ml for SIVΔ*nef* (Figure 1); the median peak load being significantly lower in SIV-rtTAΔ*nef* -infected macaques (ρ = 0.001, Mann–Whitney rank sum test). Otherwise, in general, plasma vRNA load kinetics followed a typical attenuated pattern with an acute peak typically 14 days post infection (p.i.); although occasionally as late as 28 days p.i. followed by a rapid decline with vRNA dropping below the limit of detection between 42 and 84 days p.i. and remaining undetectable or with transient peaks of <120 vRNA/copies/ml thereafter. SIV-rtTAΔ*nef* -infected macaques E65 and E70, both with delayed peak viraemia (28 days p.i.), had relatively high “set points” of >100 vRNA copies/ml, although in E65, levels had declined by 140 days p.i. Surprisingly however, despite withdrawal of dox from 175 days p.i., two transient peaks of plasma viraemia were detected in this animal. Macaque E74, infected with SIVΔ*nef*, exhibited a high set-point of approximately 10^3^ copies/ml and macaque E76, despite an initial decline in vRNA, displayed a progressive rebound of plasma vRNA.

PCR analysis of PBMC revealed that, with one exception (E61), infected macaques had a peak of proviral DNA 14- 22 days p.i. regardless of the infecting virus; however, the median peak frequency of proviral DNA was significantly lower (ρ < 0.001, Mann Whitney rank sum test) in macaques infected with SIV-rtTAΔ*nef* (4.38 copies/10^5^ cell equivalents) compared with animals infected with SIVΔ*nef* (371copies/10^5^ cell equivalents) (Figure 1). Following withdrawal of dox from Group A, proviral DNA was undetectable in 3 macaques but detected transiently and at <5 copies/10^5^ cell equivalents in the remaining 3 SIV-rtTAΔ*nef* -infected macaques.

Comparison of peak and day 175 plasma vRNA and proviral DNA concentrations showed no statistical differences between Groups A and B (vRNA peak: ρ =0.31; vRNA d175: ρ =0.94; proviral DNA peak: ρ =0.39; proviral DNA d175: ρ =0.31, Mann–Whitney rank sum test) indicating no significant differences in virological changes between these groups of animals before cessation of dox.

### Differential localisation of SIV-rtTAΔ*nef* and SIVΔ*nef* in tissues

Two animals from each group were killed humanely 230 days p.i. (Group A; 56 days after withdrawal of doxycycline) or 160-167 days p.i. (Groups B & C) for analysis of bio-distribution and T cell composition in small intestine. Proviral DNA was detected in all tissues analysed from macaques infected with SIVΔ*nef*, generally at concentrations exceeding those in PBMC (Table 1). Likewise animals infected with SIV-rtTAΔ*nef* and maintained on dox had widely distributed proviral DNA but concentrations overall were lower than those in animals of Group C, particularly in spleen and mesenteric lymph nodes. A further reduction in proviral DNA concentration was seen in the spleens and peripheral lymph nodes of the two SIV-rtTAΔ*nef*-infected macaques from which dox had been withdrawn. Proviral DNA was not detected in the thymus of SIV-rtTAΔ*nef* -infected animals and was detected in the small intestine in only one animal, when dox was maintained. To investigate further whether persisting proviral DNA was transcriptionally active with or without doxycycline quantitative rtPCR was performed on cellular RNA isolated from spleen, PLN and MLN samples. Detectable signals between 10^2^ and 10^5^ copies per 50 ng total RNA were detected in these tissues in E74 infected with SIVΔ*nef* and a signal of 248 copies per 50 ng total RNA from E78 also infected with the same virus. Amongst macaques infected with the doxycycline dependent SIV-rtTA Δ*nef* signals just above the cut-off value of the assay (20 copies per 50 ng total RNA) were detected in selected tissues from E69 and E72 that were still receiving doxycycline. No signals above cut-off were detected in macaques infected with SIV-rtTA Δ*nef* where doxycycline had stopped being administered 56 days before the tissues were analysed.

**Table 1:** Proviral DNA loads in tissues

**Animal No.**	**SIV DNA copies */ *10**^**5 **^**MNC**					
	**SlV-rtTA∆*nef***				**SlV∆*nef***	
	**Dox withdrawn**		**Dox maintained**			
	**E62**	**E64**	**E69**	**E72**	**E74**	**E78**
PBMC	<1^a^	<1	1.37	<1	2.59	2.63
Spleen	1.79	2.47	10.3	8.02	95.0	25.83
Peripheral LN^b^	<1	1.46	12.1	88.4	50.1	50.3
Thymus	<1	<1	<1	<1	ND^c^	ND
Mesenteric LN	<1	4.93	4.61	1.92	178.2	120.3
Small intestine	<1	<1	<1	4.27	3.87	7.38
Large intestine	1.31	2.58	4.4	2.68	1.88	12.8

^a^ < indicates below the limit of detection; ^b^*LN* lymph node, ^c^*ND* not done.

**Figure 2:**
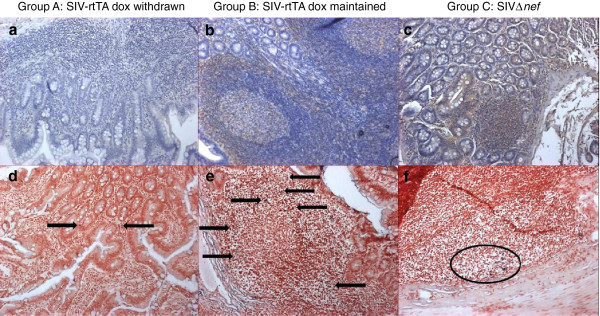
**Distribution of SIV envelope antigen and SIV RNA in small intestine.** Representative images show (**a**-**c**) immunohistochemical staining for anti-SIV envelope (brown cell surface staining) and (**d**-**f**) *in situ* hybridisation for replicating SIV (purple stained cells). Low levels of anti-envelope staining within the lamina propria/villi of Group A animals (**a**) corresponds to sites of low level viral replication (**d**, arrows). Higher levels of anti-envelope staining within the follicular marginal zones of Group B animals (**b**) and follicles of Group C animals (**c**) also correspond to sites of increased levels of viral replication (**e**, arrows; **f**, within oval). Magnification ×100.

Since the small intestine is a major site for virus replication we were interested to determine bio-distribution in this tissue. Haematoxylin and eosin staining revealed no gross pathology regardless of virus status (data not shown). Next, sections of small intestine from each animal were examined by immunohistochemistry using an SIV-Env specific monoclonal antibody for the presence of SIV envelope (Figure 2a-c). Group A macaques had very low levels of envelope detection throughout all regions of the small intestine including Peyer’s patches. By contrast Group B & C macaques demonstrated moderate levels of envelope detection within Peyer’s patches and associated germinal centres, extending to nearby crypts in Group C animals.

The presence of SIV-infected cells within the small intestine was examined by *in situ* hybridisation using a cocktail of three probes complementary with SIV RNA transcripts (Figure 2d-f). Whilst SIV positive cells were detected within the small intestine of all animals the numbers and positioning of these cells differed between groups. Sections of small intestine from Group A animals contained very low levels of SIV productively-infected cells within T cell areas of the lamina propria. Analysis of sections from Group B and Group C small intestine demonstrated the presence of increased numbers of SIV positive cells within Peyer’s patches with those from Group B animals being present largely within the marginal/mantle zones surrounding secondary follicle germinal centres and those within Group C animals being mainly clustered within the germinal centres themselves.

### Active replication of attenuated SIV reconfigured the composition of the memory T cell compartment

**Figure 3:**
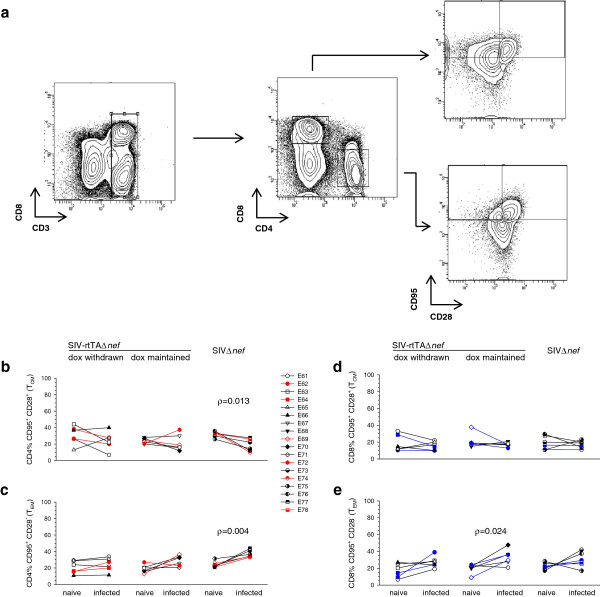
**Analysis of peripheral blood T**_**CM **_**and T**_**EM **_**populations.** Memory subsets were defined on CD28 expression within the CD95^+^ T cell population before and after infection under different conditions of replication permissivity. (**a**) Representative result illustrating the gating strategy based on CD28 expression. Comparison of frequencies of global T_CM_ and T_EM_ CD4^+^ (**b** &**c**) and CD8^+^ (**d** &**e**) T cell populations before and after chronic SIV infection. Statistically significant differences are shown as ρ values determined by paired *t*-test.

To investigate the impact of infection on memory T cell phenotype, T central memory (T_CM_) and T effector memory (T_EM_) were enumerated on CD3^+^CD4^+^ and CD3^+^CD8^+^ -gated populations by staining for expression of CD95 and CD28 according to Pitcher *et al.*, [26] (Figure 3a). CD4^+^ T cells from macaques infected with SIVΔ*nef*, showed a statistically significant reduction (ρ = 0.013; paired *t*-test) in T_CM_ (CD95^+^, CD28^+^) with a concomitant increase (ρ = 0.004; paired *t*-test) in the proportion of T_EM_. A similar trend was seen in animals infected with SIV-rtTAΔ*nef* under replication permissive conditions but did not reach statistical significance; whereas, under replication non-permissive conditions, following withdrawal of dox, only random changes were seen (Figure 3b, c). In the CD8 memory T cell populations, a similar trend was seen although statistical significance was reached only in the increase in the proportions of T_EM_ in animals infected with SIV-rtTA maintained on dox (ρ = 0.024; paired *t*-test) (Figure 3d, e).

**Figure 4:**
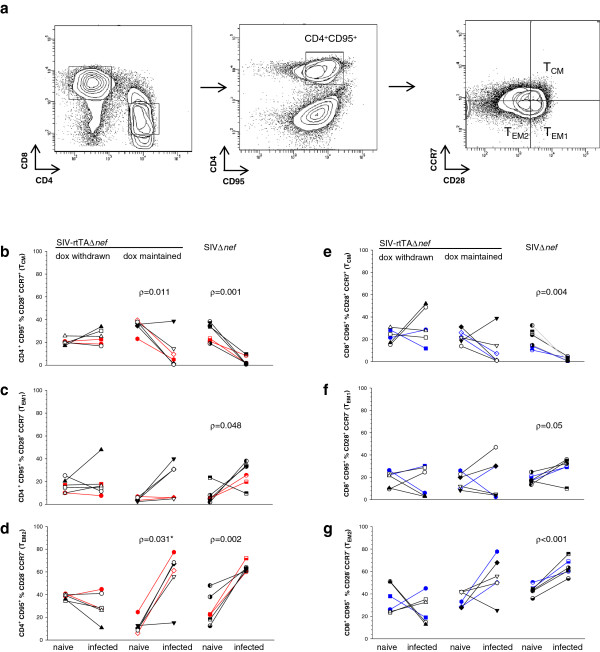
**Analysis of peripheral blood T**_**CM**_**, T**_**EM1 **_**and T**_**EM2 **_**populations.** Memory subsets were defined on differential expression of CCR7 and CD28 within the CD95^+^ T cell population before and after infection under different conditions of replication permissivity. (**a**) Representative result illustrating the gating strategy. Comparison of frequencies of global T_CM_, T_EM1_ and T_EM2_ CD4^+^ (**b**, **c** &**d**) and CD8^+^ (**e**, **f** &**g**) T cell populations before and after chronic SIV infection. Statistically significant differences are shown as ρ values determined by paired *t*-test.

Next, we determined the expression of CD28 and CCR7 on CD95^+^ CD4^+^ and CD8^+^ T cells. Thus memory cell populations were defined along the T_CM_ to T_EM_ differentiation axis according to Picker *et al*[27,28] as T central memory (T_CM_) CD28^+^CCR7^+^; transitional effector memory (T_EM1_) CD28^+^CCR7^-^ and fully differentiated effector memory (T_EM2_) CD28^-^CCR7^-^. Both SIV-rtTAΔ*nef* -dox-maintained and SIVΔ*nef* -infected animals, displayed a strong polarisation to a global CD4^+^ T_EM2_ phenotype, with a corresponding significant reduction in T_CM_ frequency (Figure 4). Similar trends were observed in the CD8^+^ T cell populations; although statistical significance was reached only in SIVΔ*nef* -infected animals. In addition a significant polarisation toward T_EM1_ was seen in both CD4 and CD8 cells from animals infected with SIVΔ*nef* with only animal E77 being an exception; whereas, only a proportion of infected-animals of group B had an increase in this subset. Of particular note, global polarisation toward T_EM2_ was not evident in animals of Group A, indicating that replication-permissive conditions were necessary for the maintenance of this phenotype.

To investigate whether SIV- or dox-induced translocation of microbial products from the gut may be driving the extensive T cell polarisation observed, LPS and sCD14 were quantified by ELISA in plasma from naïve dox-treated, SIV-rtTAΔ*nef*, SIVΔ*nef* and wild-type SIV-infected animals. LPS was undetectable and concentrations of sCD14 were close to undetectable in all animals except those infected with wild-type SIV (Additional file 1: Figure S1). Furthermore, the proportions of CD4^+^ and CD8^+^ T cells with effector memory phenotype in the blood of these animals fell within the ranges measured in non-dox treated naïve macaques.

### Viral replication imprinted expression of the gut homing marker α4β7 on CD95^+^ T cells

**Figure 5:**
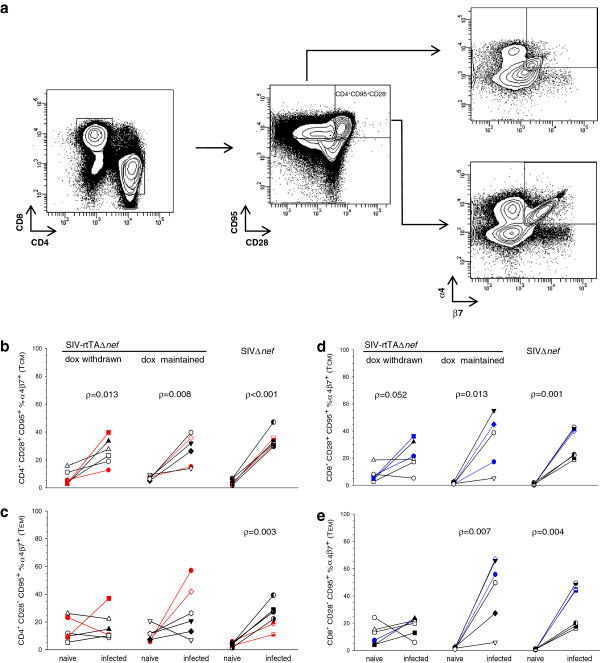
**Analysis of peripheral blood gut-homing α4**^**+**^**β7**^**+ **^**T**_**CM **_**and T**_**EM **_**populations.** Subsets were defined on expression of CD28 within the CD95^+^ T cell population before and after infection under different conditions of replication permissivity. (**a**) Representative result illustrating the gating strategy. Comparison of frequencies of global, gut-homing, T_CM_ and T_EM_ CD4^+^ (**b** &**c**) and CD8^+^ (**d** &**e**) T cell populations before and after chronic SIV infection. Statistically significant differences are shown as ρ values determined by paired *t*-test.

Because the gut associated lymphoid tissue (GALT) is a primary site of SIV and HIV replication regardless of the viral route of entry [29] and the α4β7 integrin, specifically expressed on gut-homing T cells, is upregulated in SIV/HIV infection [30] and may play an important role in pathogenesis, we were interested to determine the effect of viral replication status on α4β7 subsets. Infection with either SIV-rtTAΔ*nef* or SIVΔ*nef* induced α4^+^β7^+^ CD4^+^CD95^+^ and CD8^+^CD95^+^ T_CM_ and T_EM_ subsets although the change in CD4^+^ T_EM_ cells failed to reach statistical significance the result was skewed by macaque E67, which also was an exception in the CD8^+^ T_EM_ population (Figure 5). Interestingly, while active viral replication was necessary to maintain α4^+^β7^+^ T_EM_ in both CD4 and CD8 populations, α4β7 expression was imprinted on CD4 and CD8 T_CM_ from the majority of animals infected with SIV-rtTAΔ*nef* from which dox had been withdrawn for 56 days *i.e.* under non-permissive conditions for viral replication.

### Gut-resident T cells from macaques with actively replicating SIV were polarised toward T_EM2_ but less so than were circulating T cells

Next, we analysed T cell phenotypes in MNC isolated from the small intestines of two animals from each group at necropsy together with cells from two uninfected, dox-dosed animals as controls. There was no evidence of SIV-induced reduction in the proportion of CD4^+^ cells in the small intestine with the possible exception of E78 (infected with SIVΔ*nef*); although higher frequencies of CD8^+^ T cells were observed in all SIV-infected animals compared with controls. Interestingly, the profound global polarisation toward T_EM1_ and particularly T_EM2_ phenotype seen in the blood of animals under replication permissive conditions was less apparent in the small intestine. Nonetheless, the overall trend was similar with respect to the fully matured T_EM2_ phenotype but was not evident in the intermediate T_EM1_ populations of CD4^+^ and CD8^+^ cells (Table 2).

**Table 2:** Immunophenotype of small intestinal MNC (%)

**Animal No.**	**Uninfected controls**		**SIV-rtTA**				**WT-SIV∆*nef***	
	**Dox maintained**		**Dox withdrawn**		**Dox maintained**			
	**J9**	**J1O**	**E62**	**E64**	**E69**	**E72**	**E74**	**E78**
CD4	47.7	44.6	55.1	54.9	50.3	43.7	50.0	39.3
CD4^+^CD95^+^CD28^+^CCR7^+^	37.9	65	57.6	66.5	31.6	37.9	22.8	ND
CD4^+^CD95^+^CD28^+^CCR7^-^	27.2	13.8	13.4	9	26.4	6.2	5.2	ND
CD4^+^CD95^+^CD28^-^CCR7^-^	17.2	10.9	9.3	16.7	29	29.6	26	ND
CD8	26.8	28.7	44.0	43.1	48.9	49.8	45	51
CD8^+^CD95^+^CD28^+^CCR7^+^	36.2	43.8	32.7	36.4	19.7	26.7	22.8	ND
CD8^+^CD95^+^CD28^+^CCR7^-^	16.8	21.7	17.5	24.8	16.6	1.7	12.9	ND
CD8^+^CD95^+^CD28^-^CCR7^-^	26.2	18.9	34.4	27.7	40.5	45.3	36.3	ND

*ND* not done.

### Total polyfunctionality of SIV-specific peripheral T cells was associated with replication permissivity but group-specific cytokine signatures were not evident

**Figure 6:**
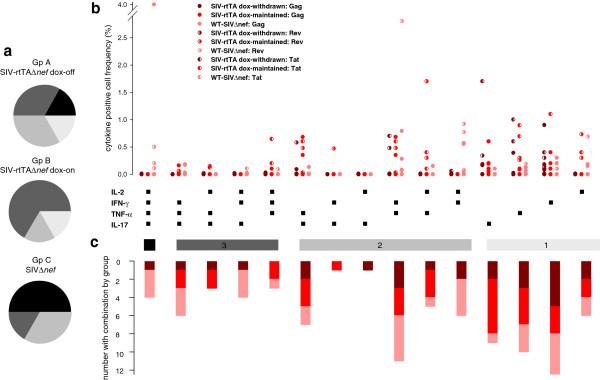
**Intracellular cytokine staining of SIV-specific CD4**^**+ **^**peripheral blood T cells.** Cell populations were determined by multiparametric flow cytometry. All analyses were performed at the end of the experimental infection schedule for each group of SIV-infected macaques. Pie charts show the proportion of animals in each group with maximum polyfunctionality regardless of which SIV peptide pool was recognised (**a**). Maximum polyfunctionality (4 parameter) is shown in black and descending degrees of polyfunctionality in shades of grey. Scatter charts show the frequency distribution for each SIV peptide pool (different symbols) and the degree of polyfunctionality for animals of each group (different colours) (**b**). Group A is shown in dark red, Group B in red and Group C in pink. Stacked bars show the number of animals in each group of 6 animals responding with each combination of cytokines (**c**).

**Figure 7:**
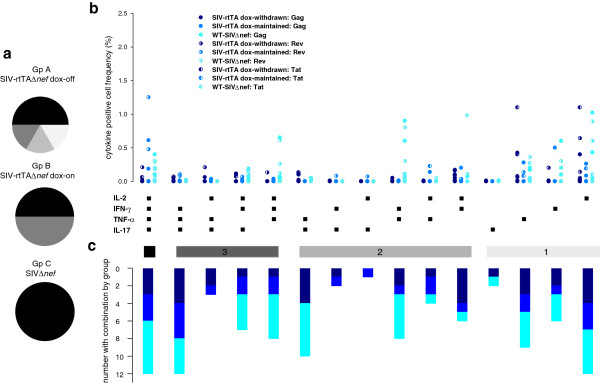
**Intracellular cytokine staining of SIV-specific CD8**^**+ **^**peripheral blood T cells.** Cell populations were determined by multiparametric flow cytometry. All analyses were performed at the end of the experimental infection schedule for each group of SIV-infected macaques. Pie charts show the proportion of animals in each group with maximum polyfunctionality regardless of which SIV peptide pool was recognised (**a**). Maximum polyfunctionality (4 parameter) is shown in black and descending degrees of polyfunctionality in shades of grey. Scatter charts show the frequency distribution for each SIV peptide pool (different symbols) and the degree of polyfunctionality for animals of each group (different colours) (**b**). Group A is shown in dark blue, Group B in blue and Group C in cyan. Stacked bars show the number of animals in each group of 6 animals responding with each combination of cytokines (**c**).

Having established that replication permissive conditions were associated with driving the peripheral and to some extent the small intestinal global T cell population toward a T_EM2_ phenotype in both the CD4 and CD8 compartments, we sought to determine whether replication permissivity influenced the quality and quantity of SIV-specific T cells. Following stimulation *in vitro* with SIV Gag, Rev or Tat peptide pools, polychromatic flow cytometry was employed to analyse intracellular staining for IFN-γ, IL-2, TNF-α, and IL-17. First, we determined the proportion of macaques in each group showing maximum polyfunctionality, regardless of frequency or individual antigen specificity. Thus, for CD4^+^ T cells 50 percent of the SIVΔ*nef*-infected animals exhibited the highest degree of polyfunctionality, whereas 66.6% of animals infected with SIV-rtTAΔ*nef* maintained on dox had a maximum of 3 functions and 1 of 6 and 2 of 6 animals infected with SIV-rtTAΔ*nef* from which dox had been withdrawn had 4 or 3 functions respectively (Figure 6a). For CD8^+^ T cells, all animals of Group C had polyfunctional cells, 50% of Group B animals had 4 functions and the remaining animals had 3 functions and 50% of animals from Group A had 4 functions but the remaining animals each had 3, 2 or 1 functions (Figure 7a). Taken together, these results indicate that the likelihood of exhibiting SIV-polyfuctionality with respect to the cytokines analysed was associated with the replicative capacity and status of the infecting virus.

Next, we sought to determine whether SIVΔ*nef* and SIV-rtTAΔ*nef*-infected macaques under replication permissive and non-permissive had distinguishable patterns of SIV antigen-specific T cell cytokine responses as it may be possible eventually to identify protective T cell signatures. The frequencies of SIV-specific cytokine secreting cells were analysed with respect to all combinations of cytokines for each infection group. In both CD4^+^ and CD8^+^ populations there was a high degree of animal to animal variability in cell frequency regardless of virological status (Figures 6b &7b) and a high degree of variability in the proportion of animals with reactive cells for each cytokine combination (Figures 6c &7c); however, there was a trend to higher overall polyfunctionality in CD8^+^ cells, most notably in animals under replication permissive conditions. CD4^+^ IL-17^+^ cells were found principally as single secretors, particularly in rtTA dox-maintained animals and in combination with TNF-α; whereas, CD8^+^ IL-17^+^ cells were virtually absent in all groups (Figure 7).

The relatively small sample size precluded detailed statistical analysis at the level of individual cytokine combinations and antigen specificity with respect to individual cell frequencies; therefore the data was analysed by principal component analysis with respect to CD4 and CD8 expression, frequency and proportion of SIV-specific responses to the 15 combinatorial cytokine patterns. Although several outlier individuals were evident, overall no signature pattern was recognised with respect to spatial clustering by infection group (Additional file 2: Figure S2).

### Total SIV-specific T cell frequencies were higher in the small intestine compared with the peripheral circulation and polyfunctionality was maintained under replication non-permissive conditions

**Figure 8:**
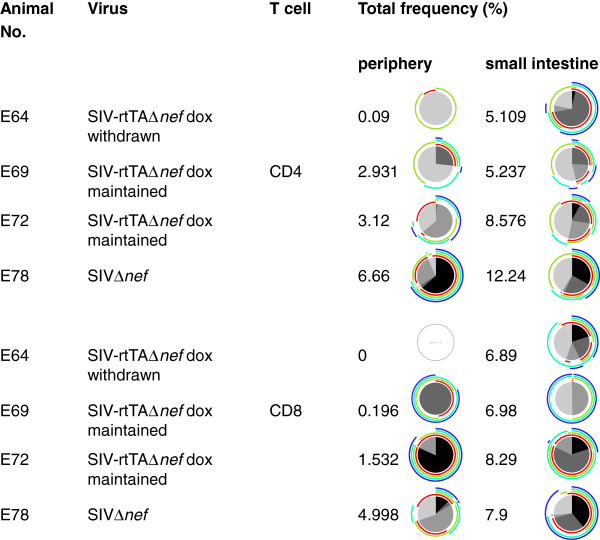
**Comparison of frequencies of SIV-specific T cells in the circulation and small intestine.** Pie charts show the proportion of cells expressing 4, 3, 2 or 1 cytokine with maximum polyfunctionality shown in black followed by shades of grey. Arcs show individual cytokine combinations. IL-2 - red; IFN gamma - green; TNF-alpha - turquoise; IL-17 - blue.

Flow cytometric analysis of intracellular cytokine stained (ICS) T cells revealed that MNC contained higher frequencies of SIV-specific T cells than were measured in the peripheral circulation of the corresponding animals (Figure 8). Sufficient cells were available from only one of two animals from Groups A and C respectively that went to necropsy examination. Nonetheless, interestingly, E64 (SIV-rtTAΔ*nef*, dox withdrawn) had few, if any, circulating SIV-specific T cells whereas CD4^+^ T cells in the small intestine were predominantly IL-2, TNF-α, IL17, and IL2, IFN-γ, TNF-α triple positive at high frequency. Likewise, CD8^+^ small intestinal T cells from E64 had a high proportion of polyfunctional cells at high frequency including ~20% expressing all 4 cytokines. Although the overall frequency of antigen-specific T cells was invariably higher in the small intestine compared with the peripheral circulation (ρ < 0.001; paired *t*-test), the proportion of polyfunctional cells and the frequency combination was variable between the two compartments. Again, it was not possible to discern any specific signature of responsiveness either between compartments or between animals from different groups.

## Discussion

We have used a novel replication-conditional SIV-rtTAΔ*nef* to compare *in vivo* the influence of infection on the global T memory cell compartment and the frequency of SIV-specific polyfunctional T cells under replication permissive and non-permissive conditions at times when, in the majority of macaques, vRNA was undetectable in the circulation. Results were compared to those obtained from macaques infected with the parental SIVΔ*nef*. The SIV-rtTAΔ*nef* construct had previously been optimised for replication in rhesus macaque PBMC through evolution *in vitro* without loss of dox-control [25]. Here we have shown that this construct is fully infectious *in vivo* in rhesus macaques and induces a similar overall pattern of plasma vRNAemia to SIV-Δ*nef*. The higher acute phase replication of the non-rtTA variant virus likely accounts for the higher proviral loads detected particularly in spleen and mesenteric lymph nodes of animals infected with this virus compared with those infected with SIV-rtTAΔ*nef*. Interestingly, lower proviral loads were detected in spleen and peripheral lymph nodes from dox-withdrawn animals, suggesting a higher turnover of SIV-infected CD4^+^ T cells in these tissues, which may therefore be primary sites of occult replication. Evidence of proviral transcription was seen in peripheral lymph nodes of macaques E69 and E72 and the spleen of E72 which were infected with SIV-rtTAΔ*nef* and maintained on dox. Similar activity was absent in the two macaques sampled from which dox was withdrawn; however, this analysis is limited by the sample size. Interestingly, much higher levels of SIV *gag* transcripts were detected in the spleen and peripheral lymph nodes of macaque E74 and the peripheral lymph nodes of macaque E78; likely reflecting the higher replicative fitness of SIVΔ*nef*. Detection of vRNA by *in situ* hybridisation and Env antigen by immunocytochemistry in the absence of proviral DNA in the small intestines of SIV-rtTA infected animals, albeit at very low levels in dox-withdrawn animals, probably reflects the sensitivity of these techniques to detect localized pockets of infection in tissue that is otherwise free from SIV-infected cells. Relatively high levels of Env staining were observed in dox-maintained animals and in animals infected with SIV-Δ*nef* with those Env positive tissue regions not correlating to sites of viral replication likely demonstrating trapping of antigen by local antigen presenting cells including follicular dendritic cells. It is also interesting to note that sites of viral replication within the small intestine were subtly different between these groups of animals.

The reduction in peak viraemia and reduced proviral load in PBMC seen with SIV-rtTAΔ*nef* compared with SIVΔ*nef* demonstrates that despite optimisation of the virus *in vitro*, the addition of the rtTA element had a fitness-cost *in vivo*. Interestingly, two macaques infected with SIV-rtTAΔ*nef* and 2 macaques infected with SIVΔ*nef* displayed persisting plasma viraemia. We have observed a similar effect in rhesus macaques using a minimally *nef*-deleted virus [20]; however, the extent of *nef* -deletion in the constructs used here make a direct repair of the attenuating lesion impossible. Surprisingly, transient low level plasma vRNA was detected in one SIV-rtTA-infected macaque (E65) after withdrawal of dox. Interestingly, this animal displayed persistence of plasma vRNAemia at >10^2^ copies ml^-1^ for over 100 days p.i. One possible explanation could be that a compensatory mutation for viral transactivation may have arisen in progeny virus within this animal; however, only minor changes, which do not affect the dox control were found in the LTR promoter and rtTA regions (data not shown). Very low levels of vRNA and Env protein were detected in the small intestines of dox-withdrawn animals which could be due to basal activity of the LTR promoter in the absence of dox. This promoter lacks a functional TAR element but contains the natural NF-Kappa-B and Sp1 binding sites, which are not sufficient to drive viral replication but may drive low-level gene expression [24,25]. Furthermore, in some cells the proviral LTR promoter may be activated by transcriptional enhancer elements present at or near the site of integration.

There was no evidence of elevated serum soluble CD14 (Additional file 1: Figure S1); an indicator of loss of intestinal epithelium integrity reported in chronic pathogenic infection with HIV and wild-type SIV [11,31]. Neither was there evidence of histopathological changes in the gut of those animals sampled, including one animal with persistent viraemia. Nonetheless, within the global circulating CD95^+^ T cell population there was a striking polarisation toward an effector memory phenotype in animals where virus replication was permissible. This result is consistent with data showing that SIVΔ*nef*-infection pushes the SIV-specific CD4 T cell response toward T_EM_[32]; however, unexpectedly, the skewing effect described here applies to the global CD4 population, regardless of antigen specificity, and was also seen in the CD8^+^ T cell population, albeit to a lesser extent. This polarising effect was particularly apparent when the CCR7 receptor, important for secondary lymphoid tissue homing, was used to differentiate T_CM_. Although macaques in group A had a higher baseline proportion of CD4^+^ T_EM2_ than those of Group B, these values fell within the range for all naïve macaques including those of Group C. Given the extent of the polarising effect seen in Groups B and C the pre-existing baseline values in Group A were unlikely to have influenced the result obtained after infection but this cannot be absolutely ruled out. Since only a small proportion of T cells are infected with SIV, it is evident that the skewing of immunophenotype must be acting on bystander cells, either through SIV-encoded soluble products such as Env and Tat, through SIV-encoded products released from dead and dying cells or from mediators arising as a result of SIV-induced dysregulation of lymphoid system homeostasis. Whatever the exact mechanism, which is likely to be multifactorial, our data suggest that on-going virus replication, albeit occult, is required to maintain T_EM_ polarisation. It is likely that there is a critical threshold of replication required to drive perturbation of the global T memory phenotype as (1) the effect is strongest, extending to both CD4^+^ and CD8^+^ T cells in macaques infected with SIVΔ*nef*,; (2) not all macaques infected with SIV-rtTAΔ*nef* demonstrated the majority effect in the presence of dox *i.e.* macaque E68 had increased CD4 T_EM1_, no significant increase in T_EM2_ and no proportionate loss of T_CM_ and displayed similar trends in CD8^+^ memory T cells and (3) despite the transient detection of plasma vRNA under dox-withdrawal in macaque E65 this animal did not demonstrate significant perturbation of memory phenotype while macaque E66 demonstrated a skewing toward an increased frequency of CD4^+^ T_EM1_.

SIVΔ*nef* induces α4β7^+^ gut mucosal-homing CD8^+^ T lymphocytes [30] and more recent studies with HIV have shown that α4β7 on CD4^+^ T cells may have a central role in pathogenesis through interaction with gp120 and signalling [33]. Moreover, α4^+^β7^hi^ memory T cells are preferentially infected during acute pathogenic SIV infection [34] and blocking α4β7 during acute infection decreases local and plasma virus loads [35]. In the study described here, analysis of expression of α4β7 on CD28^+^ and CD28^-^ populations of CD95^+^ CD4 and CD8 T cells showed that SIV infection in the majority of animals significantly increased the proportion of mucosal homing cells. Interestingly this effect appeared less dependent upon on-going occult virus replication for maintenance of CD95^+^ CD28^+^ T cells expressing α4β7 suggesting that these cells may be long-lived. In contrast, animals infected with non-attenuated SIVmac239 failed to show elevated proportions of CD95 CD28^+^ α4^+^β7^+^ CD4 cells, although the proportions of α4^+^β7^+^ T_CM_ CD8 cells were increased (data not shown). This observation is consistent with α4β7 expression on circulating CD4^+^ T cells being a surrogate marker of CD4^+^ T cell loss in the gut [36]. Indeed the proportion of CD4^+^ small-intestinal MNC were not decreased in attenuated SIV-infected macaques, even in animal E74 with a persistent plasma viraemia; whereas, the proportion of CD8^+^ cells was elevated. Furthermore the relatively low proportions of small intestinal CD4^+^ T cells and high proportions of CD8^+^ T cells in uninfected animals is similar to results reported recently [37]. It is interesting that the replication-dependent T_EM_ polarisation seen in the peripheral circulation was less pronounced in the GALT and was confined to the terminally differentiated T_EM2_ population; however, the small sample size limits interpretation of this observation.

Antigen-specific polyfunctional CD8^+^ T cell responses have been associated with positive control of immunodeficiency virus infection, for example in HIV-1 long term non-progressors [38]; however, the extent to which these types of responses are important in the SIV live-attenuated vaccine model is less clear. Also the relative role of polyfunctional CD4^+^ T cell responses needs further investigation. Infection of macaques with the novel SIV-rtTAΔ*nef* as described here may help to address these issues. The finding that overall there were no consistent differences in the patterns of cytokine combinations in CD4^+^ and CD8^+^ circulating T cells between the groups of macaques in the present study suggests that the repertoire of T cell polyfunctionality in the circulation is determined whilst virus is under replication-permissive conditions and was not overtly influenced by the lower peak viraemia observed in rtTA-infected animals. Although, only selected regions of the viral genome were examined in this study at a single time point, it is unlikely that significant differences between the groups would be missed. Interestingly IL-17-producing CD4^+^ and CD8^+^ cells were present in the gut and peripheral circulation despite their reported paucity following infection with virulent SIVmac [39,40] reflecting the attenuated nature of the viruses used in this study. It was notable that, in the small number of macaques studied, the polyfunctionality of SIV-specific T cells was notably different in individual animals when comparing systemic and gut mucosal responses possibly reflecting regional diversification of the T cell immune response. The lack of particular signatures of T cell polyfunctionality in the macaques may reflect the use of outbred animals where there is greater plasticity of immune responsiveness than seen in inbred species, such as mouse strains, frequently used for immunological analysis of functional correlates in infection.

## Conclusions

Overall, this study has shown that live attenuated SIV infection drives a global polarisation of T cell memory phenotype, particularly in the CD4 population toward effector memory. Moreover, maintenance of this effect likely requires on-going replication that is largely undetectable in the circulation. Although replication non-permissive conditions were associated with loss of perturbation of circulating T_EM_, these conditions had little, if any, effect on T cell polyfunctionality, at least with respect to the cytokines examined. It will now be important to determine whether these changes in T cell subsets influence the ability of animals infected with live attenuated SIV to resist superinfection with virulent virus thus informing rational design of vaccines against HIV. Recent findings in macaques vaccinated with recombinant rhesus cytomegalovirus expressing SIV genes strongly suggest that SIV-specific T_EM_ are crucial to early control of SIV following challenge [41]. Moreover, whilst this paper was in preparation, it has been reported that lymph node effector-differentiated T cell responses predict the efficacy of live attenuated SIV vaccines [42]. Further work is required to formally address the absolute requirement for dox *in vivo,* particularly with respect to the possibility of low-level basal transcription. The SIV-rtTAΔ*nef* construct should be useful to address issues such as whether constant or intermittent tissue associated virus replication is necessary to maintain T_EM_ frequency at protective levels.

## Methods

### Animals and viruses

UK captive-bred rhesus macaques (*Macaca mulatta*) of Indian origin, aged between 38 & 42 months at the start of the experiment, were housed and maintained in accordance with the Home Office (UK) Code of Practice (1998). The study was approved by the NIBSC Ethics Committee. Animals were sedated with ketamine hydrochloride prior to procedures. Plasma concentrations of dox were monitored *ex vivo* using a previously described assay [43].

Stocks of SIVmac239Δ*nef* (SIVΔ*nef*) and SIV-rtTAΔ*nef* were grown on CEMX174 cells and stored at -70°C as cell-free supernatants. The construction and optimization of SIV-rtTAΔ*nef* containing the rtTA-V16 variant with increased dox-sensitivity and activity was described previously [24,25,44]. Viral titres were determined using C8166 cells. Twelve macaques receiving 100 mg dox daily *per oral* were inoculated intravenously with 10^4^ TCID_50_ SIV-rtTAΔ*nef*. After175 days dox was withdrawn from six of the animals (Group A). Animals maintained on dox for the duration of the experiment were designated Group B. A further 6 animals were inoculated intravenously with 10^4^ TCID_50_ SIVΔ*nef* (Group C). All animals were sampled regularly for peripheral blood.

### Quantification of SIV RNA and proviral DNA

Quantitative real-time RT-PCR targeted to SIV *gag* was used to determine plasma viral load as described previously with a limit of detection of 50 SIV RNA/genome equivalents copies per ml [45]. Tissue samples were digested with proteinase K and phenol/chloroform following standard protocols according to [46]. After DNA extraction, qPCR was performed using Taqman Universal PCR Master mix (Applied Biosystems) and primers and complementary probes with sequences located in conserved *gag* regions as reported for the qRT-PCR assay. SIV proviral DNA concentrations were calculated using the Mx3005P software, expressed as copies of SIV DNA per 10^5^ PBMC/mononuclear cells (MNC). A single SIV DNA copy determined the limit of assay detection, as determined by Poisson statistics and as previously reported [46].

### Mononuclear cell isolation from peripheral blood and small intestinal tissue

Peripheral blood mononuclear cells (PBMC) were separated from 10 to 20 ml heparinised whole blood by Percoll gradient centrifugation. Small intestinal tissue (approximately 30 g) was cut into small pieces (1-2 cm) and digested with collagenase IV (Sigma). Tissue mononuclear cells (MNC) were isolated using Percoll density gradient. Briefly, pieces of tissues were washed in calcium and magnesium-free HBSS (Gibco) for 20 min at 37°C. Cells released in suspension were considered intra-epithelial lymphocytes (IEL). For lamina propria lymphocytes (LPL), pieces of tissue were further digested with 50 μg/ml collagenase IV and 100 μg/ml DNAase (Sigma) and passed through a mesh (70 mm pore size). The resulting material was treated with 2 mg/ml DNAase at 37°C for 30 min and MNCs were isolated using Percoll. Cells were re-suspended in FCS/RPMI for analysis. Due to low cell yields, IELs and LPLs from small intestinal tissue were pooled for analysis.

### Immunohistochemical and *In situ* Hybridisation Analyses

Representative sections of lymphoid tissues and small intestine were collected *post mortem*, fixed in 10% formal saline and embedded in paraffin wax using standard histological procedures. Four micron sections were cut and mounted on poly-L lysine coated slides. Prior to any treatment sections were de-waxed in xylene and re-hydrated via graded ethanol:water solutions. Sections were then stained with haematoxylin and eosin using standard histochemical procedures. *In situ* hybridisation for the detection of SIV RNA transcripts was performed with digoxigenin (dig; Roche, Lewes, UK) labelled single stranded DNA probes [47] in a cocktail containing either three probes normal to or three probes complementary with SIV transcripts using a BondMax automated staining machine, utilising the Research Mode option for protocol design and execution (Leica Microsystems USA) [48]. Quantification of ISH positive cells was performed by manually counting all positive cells within up to 10 random fields of view (×10 lens and ×10 eyepiece magnification; equivalent to 2.2 mm^2^) and data calculated as the mean number of positive cells per mm^2^.

Immunohistochemical analyses were performed on a BondMax automated staining machine. Sections were treated using a 30 min HIER1 unmasking protocol and stained using a Bond Polymer Refine staining system and protocol (Leica Microsystems, USA) incorporating an addition blocking treatment step (20 minutes at room temperature using 20% normal horse serum, 1× casein (Vector Laboratories) in 10 mM Tris/1 mM EDTA pH 7.6) prior to the addition of KK13 monoclonal antibody specific for SIVgp120 [49]. Degrees of tissue staining were graded and used to generate mean scores within each group of two animals.

### Immunophenotyping and intracellular cytokine staining

Peripheral and gut mononuclear cells were simultaneously surface stained with anti-CD3-V500 (clone SP32, BD Horizon), anti-CD4-V450 (clone L200, BD Horizon), anti-CD8-APCCy7 (clone SK1, BD Biosciences), anti-CD95-PECy7 (DX2, BioLegend), anti-CD28-PerCP-Cy5.5 (eBiosciences), anti-CCR7-FITC (R&D systems), anti-β7-APC (BD Biosciences) and anti-CD49d-PE (α4, BD Biosciences).

SIV-specific cellular immune responses were assessed using multiparametric intracellular cytokine staining (ICS) as described [50]. Briefly, thawed PBMCs were washed and re-suspended at 2 × 10^6^ cells/ml in RPMI 1640/10% FCS medium. Cells were then plated with 1 mg/ml CD49d, 1 mg/ml anti-CD28, Golgi Stop (10 ng/ml, BD), and either 5 μg/ml of 15mer SIV Gag or Tat or Rev pooled peptides (CFAR/NIBSC, Potters Bar, UK) and incubated at 37°C in a 5% CO_2_ environment with RPMI 1640/10% FCS for 14 h. PBMC were incubated with 10 ng/ml of PMA (Sigma) and 1 μg/ml ionomycin (Sigma) as positive controls. A negative control containing MNC and co-stimulatory antibodies without the peptide mix, was also included in each assay. After 2 hours, 10 ng/ml monensin (BD Biosciences) was added to all cultures. After incubation, cells were surface-stained with anti-CD4-PECy7, anti-CD8-Alexa 488 and anti-CD3 V500 (BD Biosciences), washed with PBS/2% FCS and fixed and permeabilised with Fix and Perm kit (Caltag). Intracellular cytokines were detected using anti-IFN-γ-PErCPCy5.5 (clone B27, BioLegend), anti-IL-2-PE (MQ1-17H21, ebiosciences), anti-TNF-α APC (MAB11, eBiosciences) and anti-IL-17A-Pacific Blue (BioLegend). Finally, cells were washed, fixed in PBS containing 1.5% paraformaldehyde (Sigma) and stored at 4°C prior analysis. All samples were analysed by flow cytometry (BD Canto II) within 24 h. Instrument set up and data acquisition procedures were performed according to BD Immunocytometry Systems protocols. More than 200,000 and 50,000 events respectively were acquired per sample, within the lymphocyte population of PBMC and gut-derived MNC samples. Electronic compensation was applied using antibody-capture beads (BD Biosciences) stained separately with individual monoclonal antibodies. Data were analysed using BD FACS Diva software. Lymphocyte subpopulations were identified by gating on CD3^+^ cells and subsequently delineating CD4, CD8, CD95, CCR7, CD28 and α4β7 expression. CD4 gating delineated a CD4^high^ population, by excluding weakly positive events (events located in regions of low contour density, *e.g.* reference [51]). In this way, non-conventional T cells such as NKT cells are excluded from the analysis. For the identification of T cell memory subsets, CD4^+^ and CD8^+^ cells were represented on contour plots depicting staining intensity for the CD95 and CD28 parameters. A quadrant was set according to fluorochrome/contour intensity [26] and used to discriminate between the 4 phenotypic combinations. Differential expression of CCR7 and CD28 was similarly determined using a gating protocol based on that of Picker *et al.*, [27].

To determine T cell polyfunctional cytokine responses to SIV Gag, Rev and Tat peptide pools, a combination of sequential plots were used delineating frequencies of TNF-α, IL-2, IFN-γ and IL-17 expressing cells. Background responses detected in negative control samples were subtracted for every specific functional combination. A threshold of >0.01% total CD4^+^ or CD8^+^ T cells responding after subtraction of background was used as a cut-off [52,53].

### Statistical analysis

Statistical analyses, as specified, were performed using Sigma Plot 11 (Systat Software, Inc.) and principal component analysis was used to determine any signature patterns of responsiveness (SSPS).

## Competing interests

The authors declare that they have no competing interests.

## Authors’ contributions

MM carried out flow cytometric analysis, contributed to experimental design and drafting the manuscript. NB and CH carried out the molecular virology in the animal studies and contributed to the manuscript, DF designed and carried out immunocytochemistry and ISH and provided input to the manuscript. RS participated in designing and interpreting immune-phenotyping., MR, MP and BL contributed to acquisition and preparation of biological specimens and virological results. AD and BB prepared constructs and performed sequence analysis, NA, BB, and MC conceived of the study, participated in its design and coordination. MC took overall responsibility for writing the manuscript. All authors read and approved the final manuscript.

## Supplementary Material

Additional file 1: Figure S1 Plasma concentrations of soluble CD14 in naïve and SIV-infected macaques after chronic infection. sCD14 concentrations were determined using a sCD14 ELISA (R&D Systems). Box plots show 95^th^ percentiles and median values. Dotted line shows limit of detection. Click here for file

Additional file 2: Figure S2 Analysis of multiparametric flow cytometry data for signature patterns of cytokine expression. Principal component analysis was used to determine any clustering of profiles of intracellular cytokine expression in SIV peptide-stimulated PBMC taken from macaques infected with SIV-rtTAΔ*nef* and following withdrawal of dox (replication non-permissive, Group A) or following maintenance of dox (replication permissive, Group B) or infected with non-dox dependent SIVΔ*nef* (replication permissive, Group C). PCI shows principal component scores accounting for as much variation in the original data as possible *i.e.* the transformed data from the 18 animals analysed. PCII accounts for much of the remaining variation as possible. Individual animal numbers are shown on the plot and their group designated by coloured symbols. Click here for file
