# Supplementary figures and images for: Conditionally-live attenuated SIV upregulates global T effector memory cell frequency under replication permissive conditions
Source: Retrovirology. 2013 Jun 5;10:59. doi: 10.1186/1742-4690-10-59 (PMC3706341; doi:10.1186/1742-4690-10-59)

## Slide 1
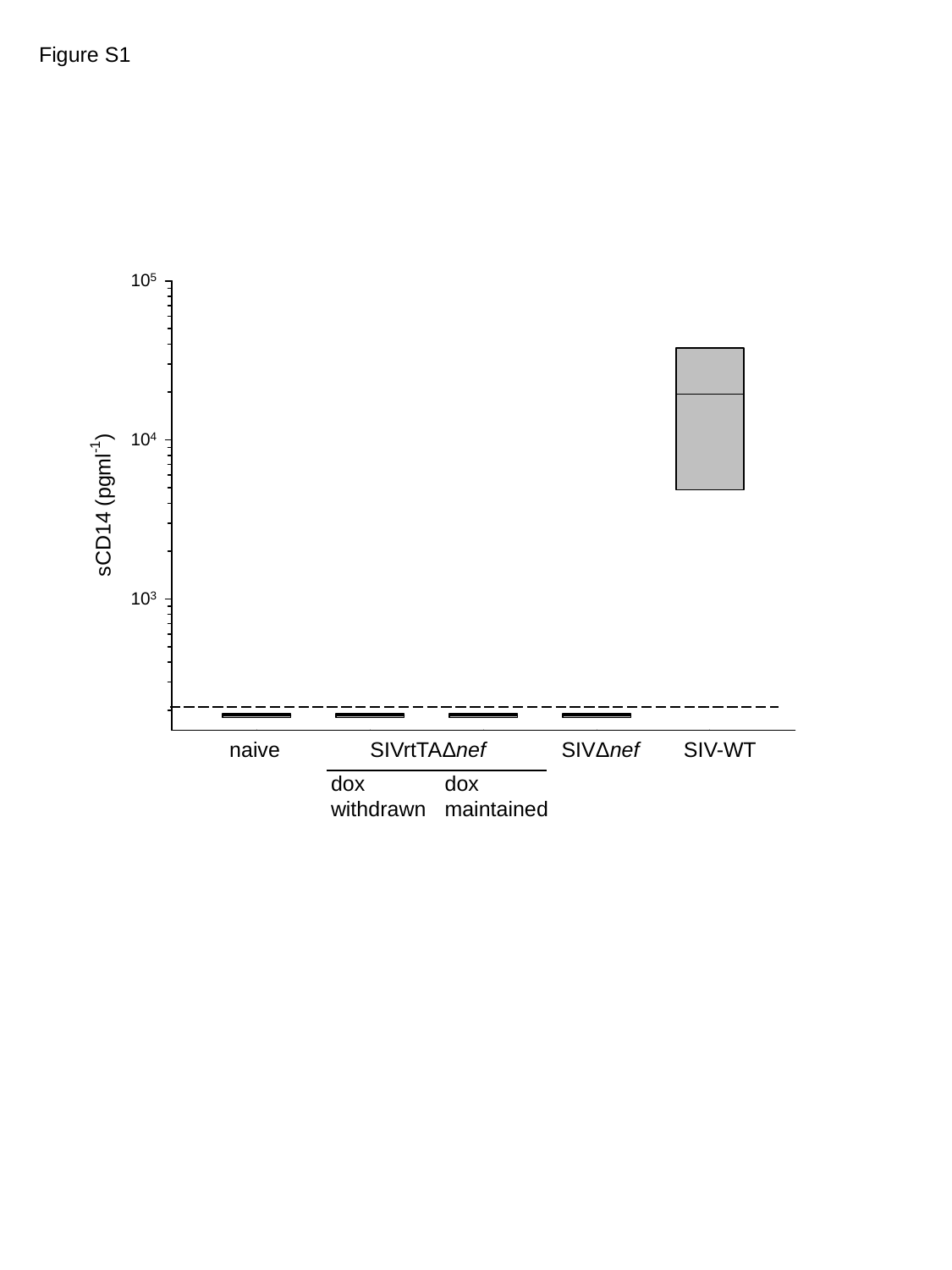

Figure S1
naive
SIVrtTAΔnef
SIVΔnef
SIV-WT
dox
withdrawn
dox
maintained

Supplement: Additional file 1: Figure S1 — Plasma concentrations of soluble CD14 in naïve and SIV-infected macaques after chronic infection. sCD14 concentrations were determined using a sCD14 ELISA (R&D Systems). Box plots show 95th percentiles and median values. Dotted line shows limit of detection. [file 1742-4690-10-59-S1.pptx]

## Slide 1
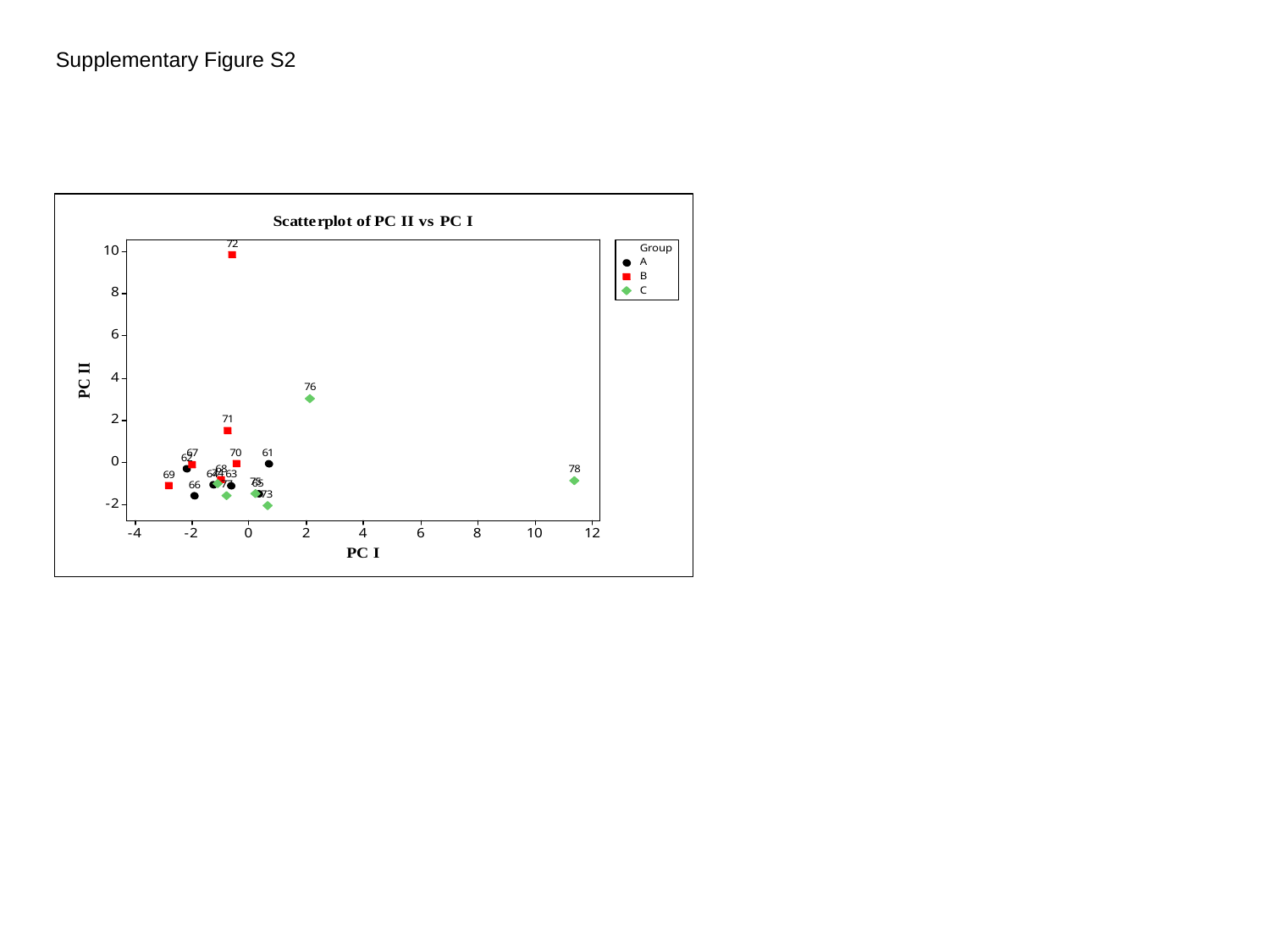

Supplementary Figure S2

Supplement: Additional file 2: Figure S2 — Analysis of multiparametric flow cytometry data for signature patterns of cytokine expression. Principal component analysis was used to determine any clustering of profiles of intracellular cytokine expression in SIV peptide-stimulated PBMC taken from macaques infected with SIV-rtTAΔnef and following withdrawal of dox (replication non-permissive, Group A) or following maintenance of dox (replication permissive, Group B) or infected with non-dox dependent SIVΔnef (replication permissive, Group C). PCI shows principal component scores accounting for as much variation in the original data as possible i.e. the transformed data from the 18 animals analysed. PCII accounts for much of the remaining variation as possible. Individual animal numbers are shown on the plot and their group designated by coloured symbols. [file 1742-4690-10-59-S2.pptx]
